# FTIR analysis of molecular composition changes in hazel pollen from unpolluted and urbanized areas

**DOI:** 10.1007/s10453-016-9445-3

**Published:** 2016-06-18

**Authors:** J. Depciuch, I. Kasprzyk, O. Sadik, M. Parlińska-Wojtan

**Affiliations:** 1 Institute of Nuclear Physics Polish Academy of Sciences, 31342 Kraków, Poland; 2 Department of Environmental Biology, Faculty of Biology and Agriculture, University of Rzeszow, Cwiklińskiej 1a, 35-601 Rzeszow, Poland; 3 Department of Chemistry, State University of New York at Binghamton, Binghamton, NY 13902 USA

**Keywords:** FTIR, Hazel, Pollen, Allergy, Air pollution, SEM

## Abstract

**Abstract:**

In this study, the effect of urbanization and environmental pollution on qualitative (structural) and quantitative changes of the *Corylus avellana* (hazel) pollen was investigated using scanning electron microscopy, Fourier Transform Infrared (FTIR) spectroscopy and curve-fitting analysis of amide I profile. The obtained spectroscopic results show significant variations in the fraction of proteins in the hazel pollen, which probably depend on various degrees of anthropopression. Our results suggest that alterations in the chemical composition of pollen, induced by urbanization and air pollutants, may intensify the allergenic potential and may cause the increase in the incidence of allergies in people. Mutations in nucleic acids are accompanied by a number of molecular changes leading to the formation of allergenic proteins. It seems that the type of habitat, where the pollen grew, affects the individual differentiation. Indeed, it was found that in the site exhibiting low pollution, the hazel pollen contain a lower amount of proteins than to the ones from a site with high anthropopression. Hence, FTIR spectroscopy and curve-fitting analysis of amide I profile can be successfully applied as tools for identifying quantitative and qualitative changes of proteins in hazel pollen.

**Graphical Abstract:**

Anthropogenic factors such as air pollution and urbanization lead to changes in structure and chemical composition of hazel pollen. Fourier Transform Infrared spectroscopy (FTIR) and Gaussian analysis showed structural changes in hazel pollen collected from sites with different absorbance values of individual chemical functional groups and changes in the secondary structure of proteins of the pollen.
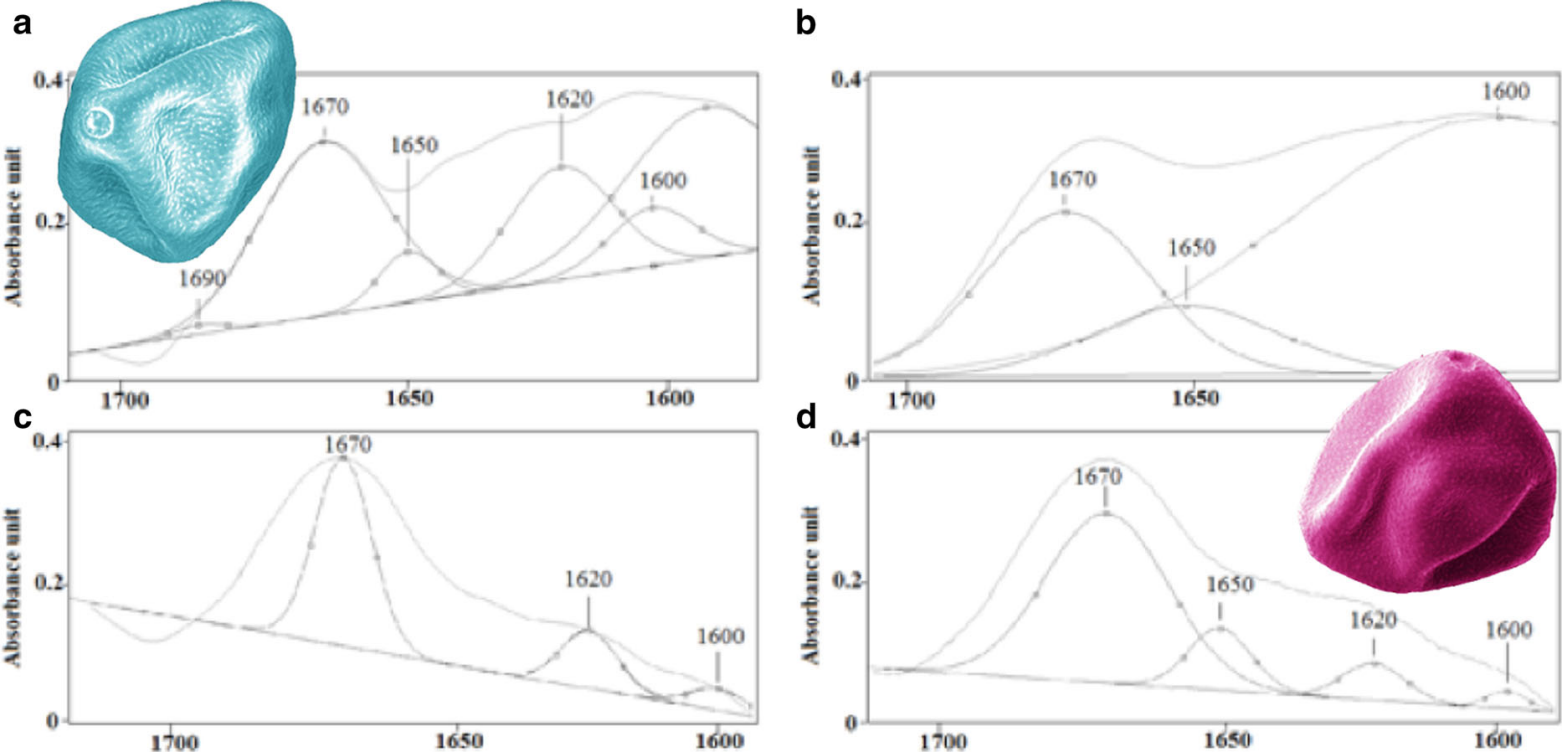

## Introduction

The prevalence of respiratory allergic diseases is increasing every year, especially in developed countries (Verlato et al. 2003; ECRHS 1996; ISAAC 1998; Sozańska et al. 2015; Timm et al. 2016). Pollen grains are recognized as important agents causing allergies in susceptible subjects, and hazel pollen is one of the most common allergen (D’Amato et al. 2007; Yalcin et al. 2013; Asam et al. 2015). As an aeroallergen, it can cause conjunctivitis, allergic rhinitis, or even asthma in sensitized individuals. In early spring, hazel pollen has been shown to be an important cause of pollinosis in Switzerland (Frei and Gassner 2008), France (Thibaudon 2003), Germany (Levy and Bircher 1994), Romania (Popescu et al. 1985), Poland (Grewling et al. 2014) and Italy (Cecchi et al. 2013). High or relatively high airborne pollen concentrations have been recorded during February–March in Italy (Longo and Sauli 2010), England (Emberlin et al. 2007), Croatia (Sikora et al. 2013) and in Poland (Nowosad et al. 2014, Kruczek et al. 2015). This period coincides with the heating season in Central Europe, when increased levels of contaminants such as SO_2_, NO_2_, CO_2_ and O_3_ are observed. Numerous studies correlating the increase in number of people suffering from allergy with morphological and biochemical changes within the pollen grain caused by air pollutants have been conducted (Emberlin et al. 1993; D’Amato 2000; Ackaert et al. 2014; Lázaro et al. 2014; Todea et al. 2013). These changes can cause a more frequent occurrence of inhalant allergies or more severe allergic reactions (Pasqualini et al. 2015; Tedeschini et al. 2015). The morphological modifications of the pollen grains can be analyzed by microscopy, which however does not provide any information about structural and biochemical changes. An alternative are molecular biology techniques, which give information about the mutation in nucleic acid of the pollen grains leading to mutations in proteins. These mutations could be responsible for structural and functional changes in peptides (Friedberg 2010; Li et al. 2010). Unfortunately molecular biology techniques are very expensive and do not provide information about biochemical and structural changes in proteins. Yet, information about structural and biochemical changes in chemical compounds building the pollen grains can be obtained by Fourier Transform Infrared (FTIR) spectroscopy. This technique, in combination with curve-fitting analysis of amide I profile, is becoming more and more common for studying biological samples. FTIR spectroscopy is fast, simple, requires small quantities of measured material, is economic and does not destroy the sample. The energy of the electromagnetic wave in the infrared range is large enough to cause vibrations of functional groups belonging to nucleic acids, proteins, polysaccharides, lipids and water, through a change in the dipole moment of bonds (Scotter 1997; Van de Voort and Sedman 1993). The FTIR technique therefore seems to be an ideal tool for measuring the chemical compounds of plant pollen of different species. The fingerprint region of FTIR spectra allows for unambiguous identification of the substances. Therefore, the FTIR technique is often used to identify the pollen species (Suphioglu et al. 1992). Indeed, there are studies, in which the authors apply spectroscopic methods for the identification of pollen and thus create spectroscopic libraries of plants and pollen (Zimmermann and Kohler 2014; Gottardini et al. 2009; Guedes et al. 2014).

The literature data indicate increasing production of allergens, due to different type of air pollutants. These factors can lead to genetic mutations and thus to changes in protein’s secondary structure (including allergenic proteins) (Guedes et al. 2014; Dell’Anna et al. 2009; Cortegano et al. 2004). The aim of this study was to determine whether anthropogenic factors such as air pollution and urbanization lead to changes in structure and chemical composition of hazel pollen. FTIR spectroscopy and curve-fitting analysis of amide I profile were applied to assess the structural changes of hazel pollen collected from sites with different forms of anthropopression.

## Materials and methods

### Materials

The *Corylus avellana* (hazel) pollen samples were collected in March 2015, in four different regions of Poland: Bieszczady National Park (natural forest-S1), Krasne village (rural area-S2), Rzeszow city (high traffic-S3) and Zawiercie city (industrial area with steel mine and iron foundries-S4). These locations differed significantly in terms of air pollution on regional and local scale, which is well shown in Table 1. The concentrations of air pollutants, mainly particulate matters and benzo(A)pirene, were the highest in cities. The pollen samples collected in the forest in the Bieszczady National Park, with the lowest air pollution, were treated as reference samples. Thus, all the samples collected from other regions were structurally and chemically compared to the ones from S1. With this experimental design, air pollution was the factor, which differentiated these locations the most. At each site, one hazel plant was chosen and then subjected to further investigation. At the beginning of hazel full flowering period, two/three inflorescence (samples) were collected from individual shrubs. Subsequently in the laboratory, the samples were dried at room temperature until pollen was released from anthers.

**Table 1:** Air quality classes and annual average concentrations of chosen air pollutants in studied sites compared to the threshold values (based on www.katowice.pios.gov.pl/monitoring/informacje/stan2014/13ocena.pdf and www.wios.rzeszow.pl/cms/upload/edit/file/opracowania/jakosc_powietrza/2014/tekst_ocena_2014.pdf)

Site	Pollutant						
	SO_2_ 350 (μg/m^3^) 1-h average	NO_2_ 200 (μg/m^3^) 1-h average	CO 10,000 (μg/m^3^) 8-h average	O_3_ AOT40 18,000 (μg/m^3^ h)	Benzo(A)pirene 1 (ng/m^3^) 8-h average	PM10 40 (μg/m^3^) annual average	PM2.5 25 (μg/m^3^) annual average
Bieszczady—S1	A; <5	A; <10	A; <400	A; 7500–8500	A; <0.6	A; <5	A; <5
Krasne—S2	A; 16–25	A; 68–85	A; 1501–2500	A; 8501–9500	A*; 1.6–2.5	A; 11–20	A; 16–20
Rzeszów—S3	A; 26–40	A; 106–135	A; 3501–4858	A; 8501–9500	C*; 3.6–4.5	A*; 21–30	C*; 21–32
Zawiercie—S4	A	A	A	C; 8628–10,534	C*	C*42	C*

A, concentrations of the pollutants below the threshold value; C, concentrations of the pollutants above the threshold value; *, concentrations of the pollutants above the threshold value mainly in heating season (December–March); AOT40, accumulated amount of ozone over of 40 ppb from May to July 2014; this threshold value is in respect to plant protection

Each pollen sample from each habitat was measured three times in order to eliminate errors and make sure that every time the spectrum looked the same. Every spectrum presented in the figures is the average of the spectra obtained for each habitat.

### SEM imaging

Scanning electron microscopy (SEM) was carried out on a FEI Quanta 3D Dual beam instrument equipped with a tungsten cathode. Directly after being collected, the hazel pollen grains were deposited the SEM stub sample holder covered with a carbon patch. Uncoated samples were imaged in low vacuum mode at 1 kV accelerating voltage using the SE detector (Everhart and Thornley 1960).

### FTIR measurements

FTIR spectra were acquired using the Vertex 70 (Bruker) spectrometer applying the Attenuated Total Reflectance (ATR) technique. The selected infrared radiation was in the average IR range (400–4000 cm^−1^). To achieve 4 cm^−1^ spectral resolution, 64 scans were used. For each pollen sample, the same absorption bands corresponding to nucleic acids, proteins, polysaccharides, lipids and water were identified. Each measurement was taken in triplicate. In order to determine the structural changes in the regions of the spectra corresponding to phospholipids, a second derivative of the spectra was calculated (Baker et al. 2014).

### Data analysis

All spectra were treated with the OPUS software. The analysis of the secondary structure of the proteins was performed by curve fitting using the GRAMS AI software from Thermo Scientific. The second-derivative spectra were calculated from the ATR-FTIR spectra after smoothing over two consecutive points. The second derivative of the FTIR spectra allows, using a mathematic algorithm increasing the resolution of the obtained FTIR spectrum (Baker et al. 2014). The resulting spectrum often has a lot of artifacts, which can be removed using the “smoothing over two consecutive points” function in the “OPUS” software. The absorption bands at low wavenumbers were free of features from water vapor, as judged from the peaks above 1750 cm^−1^. A straight baseline passing through the ordinate at 1700 and 1610 cm^−1^ was subtracted before the curve fitting. The baseline was again modified by the least-squares curve-fitting software, which allows for a horizontal baseline to be adjusted as an additional parameter to obtain the best fit. The second-derivative spectrum was used to determine the initial peak positions for curve fitting, and the peaks were fitted using Gauss functions. The area under the entire band was considered as 100 %, and each component after fitting was expressed as a percent fraction (Lahlali et al. 2014).

The concept of Fourier self-deconvolution is based on the assumption, that a spectrum of single bands, each narrow band being characteristic for a secondary structure, is broadened in the liquid or solid state. Therefore, in the amide envelope, the bands overlap and cannot be distinguished. A curve-fitting procedure can be applied to estimate quantitatively the area of each band representing a type of the secondary structure. In the pioneering work by Susi and Byler (1986), the amide I (1600–1700 cm^−1^) was deconvoluted with a Lorentzian line shape function and a resolution enhancement factor of 2.4 was applied. The deconvoluted spectrum was fitted with Gaussian shaped bands by an iterative curve-fitting procedure. The results are in good agreement with the secondary structure information obtained from X-ray crystallographic structures of the proteins under study. However, to be able to perform an analysis of best fit, in the tested fragment of the FTIR spectrum, each measurement point must have a positive value. Therefore, a “horizontal baseline” function using OPUS software was performed.

## Results

**Fig. 1:**
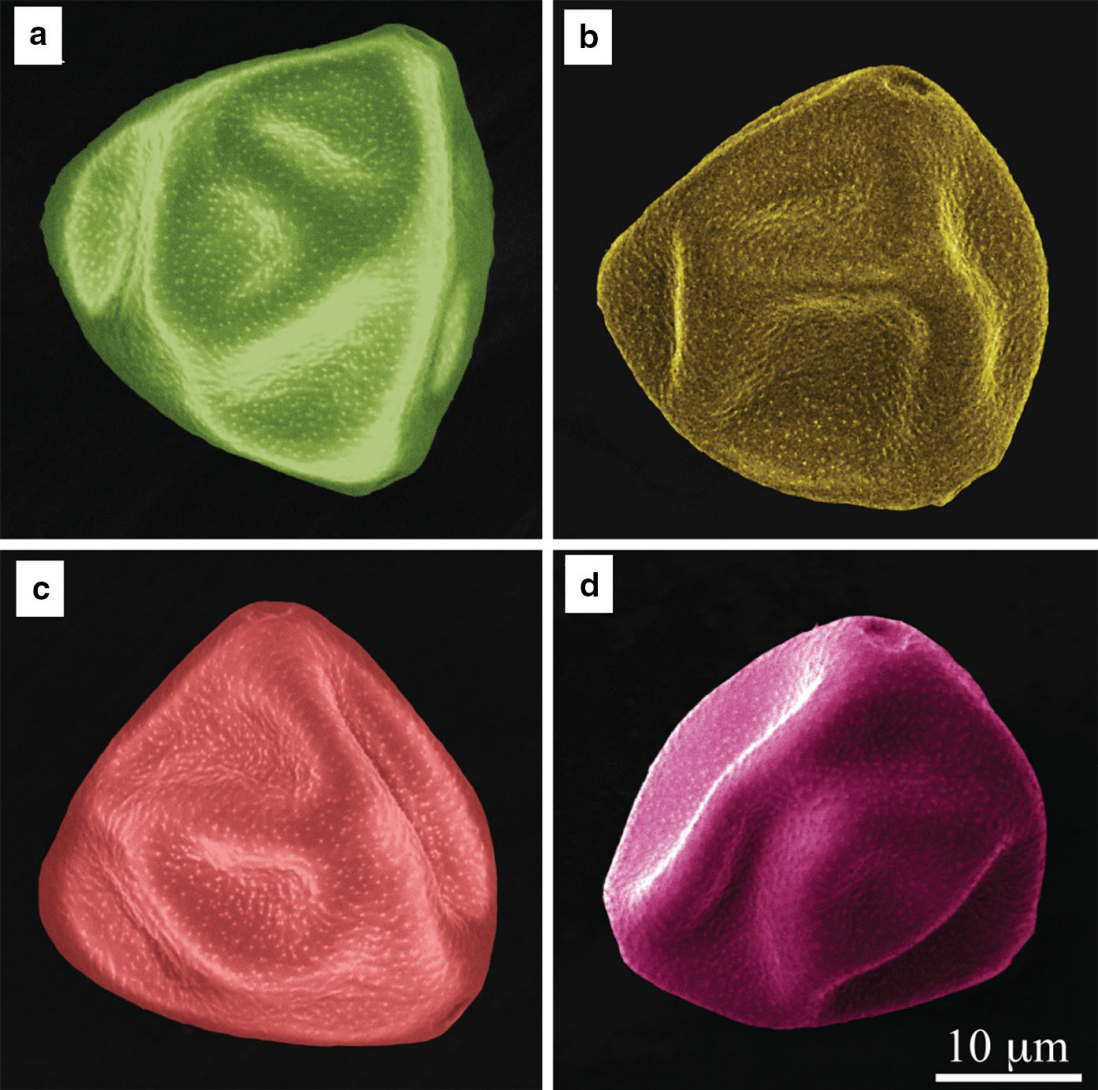
SEM images of hazel pollen grains (*polar view*) collected from: **a** Bieszczady (S1), **b** Krasne (S2), **c** Rzeszów (S3), **d** Zawiercie (S4). The *scale bar* is the same for all four photographs. The images are artificially *colored*

Figure 1 shows a set of artificially colored SEM images of the hazel pollen collected from all sites S1–S4.

**Fig. 2:**
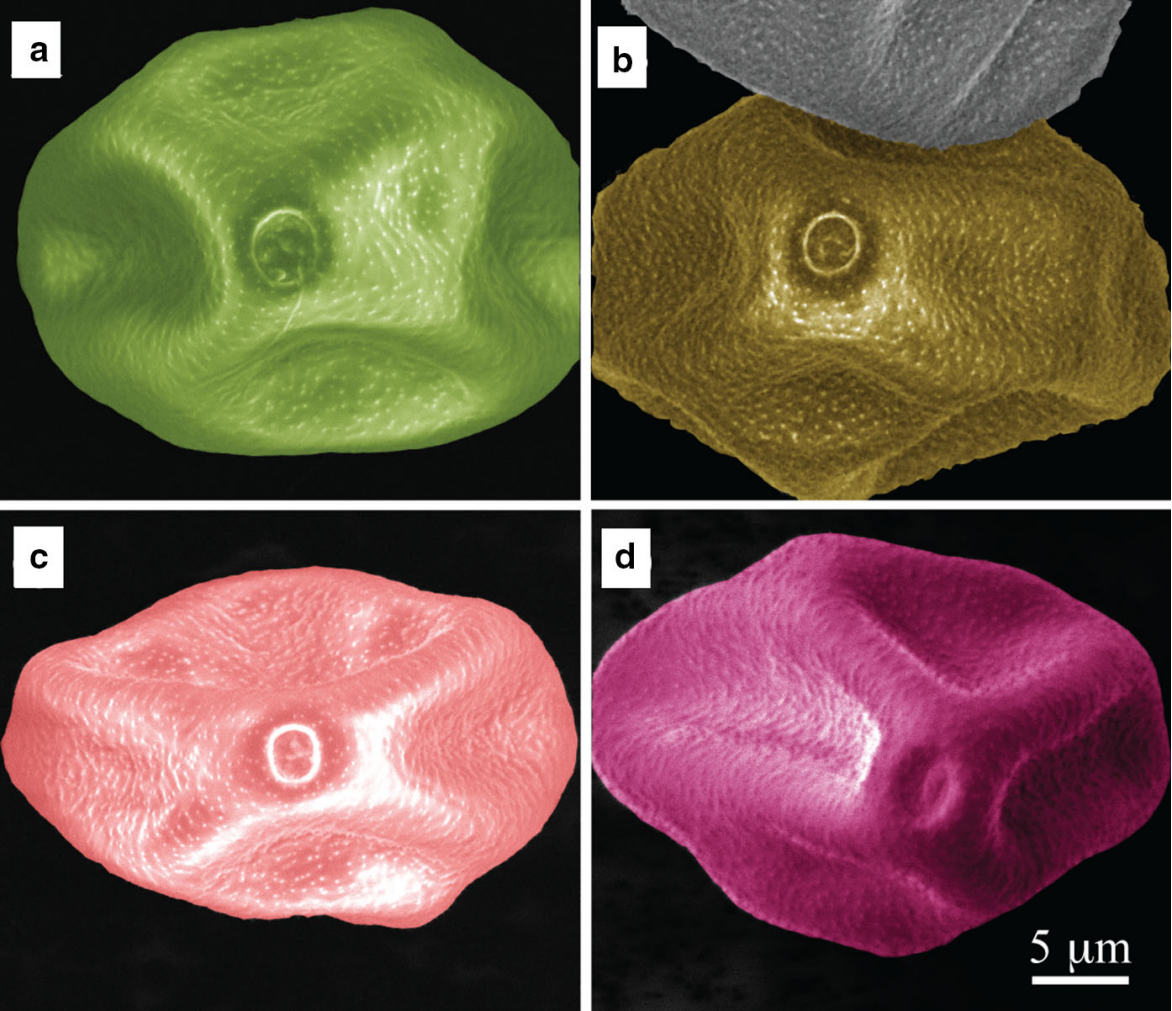
Magnified SEM images showing the pores of hazel pollen grains (*equatorial view*) collected from: **a** Bieszczady (S1), **b** Krasne (S2), **c** Rzeszów (S3), **d** Zawiercie (S4). The *scale bar* is the same for all four photographs. The images are artificially *colored*

SEM imaging did not reveal any differences in size or shape of the pollen grains collected from the four regions. The pores of the respective pollen grains are visible in their magnified SEM images shown in Fig. 2. The color code represents the four collection regions. No distinct differences in the pores morphology or their size between the respective samples could be noticed.

**Fig. 3:**
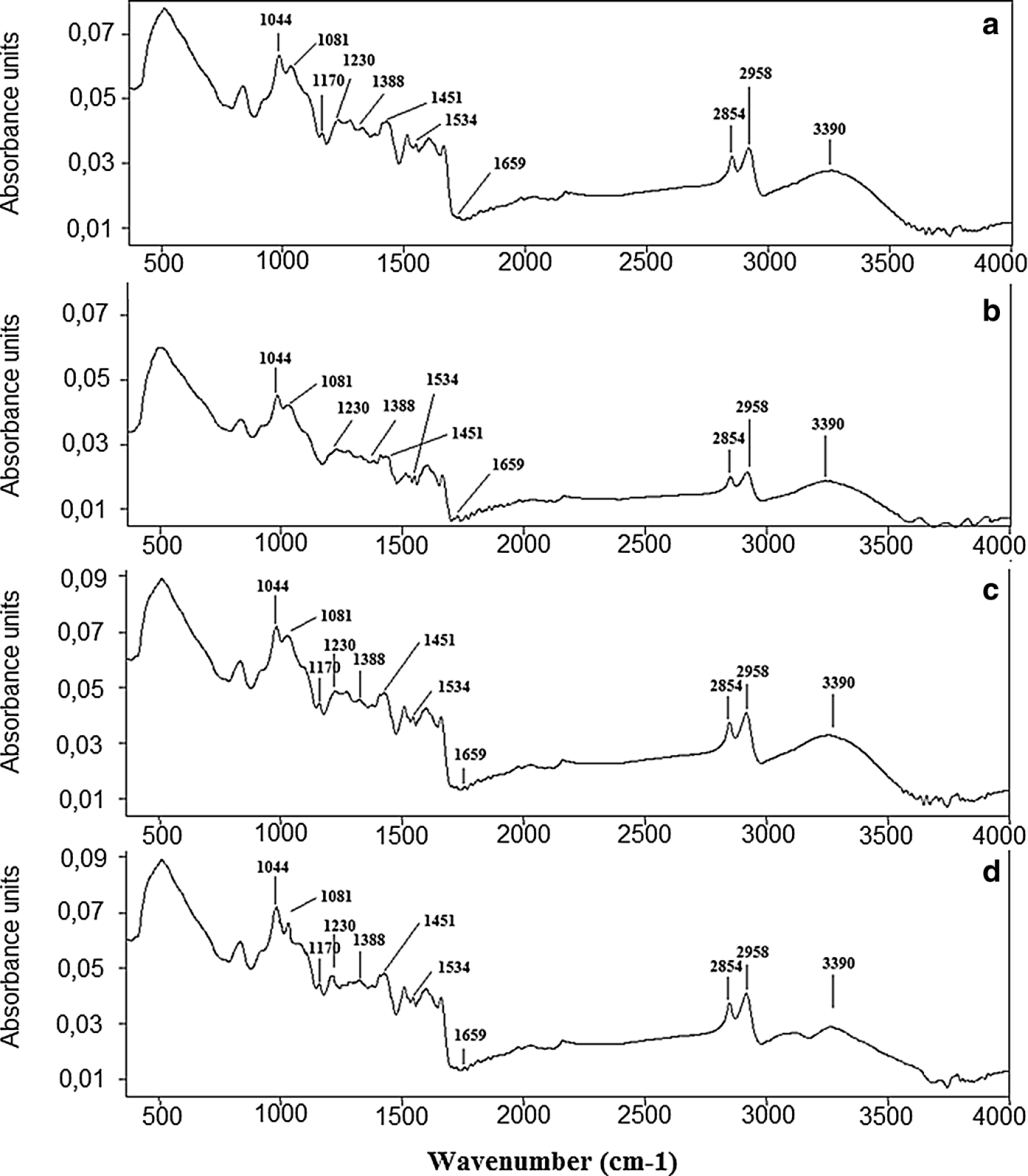
Offset FTIR spectra of hazel pollen from: **a** Bieszczady (S1), **b** Krasne (S2), **c** Rzeszów (S3), **d** Zawiercie (S4)

As SEM imaging did not reveal any differences in the pollen external structure, their chemical analysis was performed by FTIR spectroscopy to assess possible changes in the composition of their chemical compounds. Figure 3 shows the offset of the FTIR spectra indicating specific bonds for each sample. The characteristics of bonds are presented in Table 2.

**Table 2:** Values of wavenumbers with the corresponding vibrations (Armentia et al. 2002)

Wavenumber (cm^−1^)	FTIR
1044	Stretching vibration C–O group (polysaccharides)
1081	Asymmetric vibration of PO^−2^– group (nucleic acids and phospholipids)
1170	Asymmetric vibration of CO–O–C group (glycogen)
1230	Asymmetric vibration of PO^−2^– group (nucleic acids and phospholipids)
1388	Symmetric vibration of group COO– (amino acids and proteins)
1451	Bending vibration of CH_3_ (lipids)
1534	Amide II (N–H bending vibration and a stretching vibration of C–N groups)-proteins
1659	Amide I (stretching vibration of C=O)-proteins
2854	Symmetric vibration of CH_2_ group (lipids)
2958	Asymmetric vibration of CH_3_ group (lipids)
3390	Deformation vibration of OH (water)

In the FTIR spectra, specific peaks corresponding to nucleic acids, polysaccharides, proteins, lipids, water and other compounds could be identified (Fig. 3). The low wavenumber region of the FTIR spectrum originates from the chemical bonds in polysaccharides 1044 cm^−1^, nucleic acids and phospholipids (1081, 1230 cm^−1^), respectively. The peaks at 1170 and 1388 cm^−1^ originate from glycogen and amino acids. Vibrations observed in the FTIR spectrum at 1451, 2854 and 2958 cm^−1^ correspond to lipids. The higher wavenumbers in FTIR spectra are derived from proteins: 1534 and 1659 cm^−1^. The peak at 3390 cm^−1^ corresponds to water remaining in the pores of the dried pollen (Armentia et al. 2002).

FTIR spectra (Fig. 3) show that depending on the location, the chemical composition of pollen samples is different. In fact, although the measured samples are pollen of the same plant species, no two identical FTIR spectra were obtained. Indeed, it can be seen that in all four samples, the intensities of the individual peaks differ from each other, and in some spectra peaks from some functional groups are absent. The spectrum of pollen collected in S2 (Fig. 3b) does not show the presence of vibrations at wavenumber 1170 cm^−1^, which correspond to the asymmetrical vibration of the CO–OC group derived from glycogen. To obtain structural information from the samples, the second derivative from respective the peaks is calculated.

**Fig. 4:**
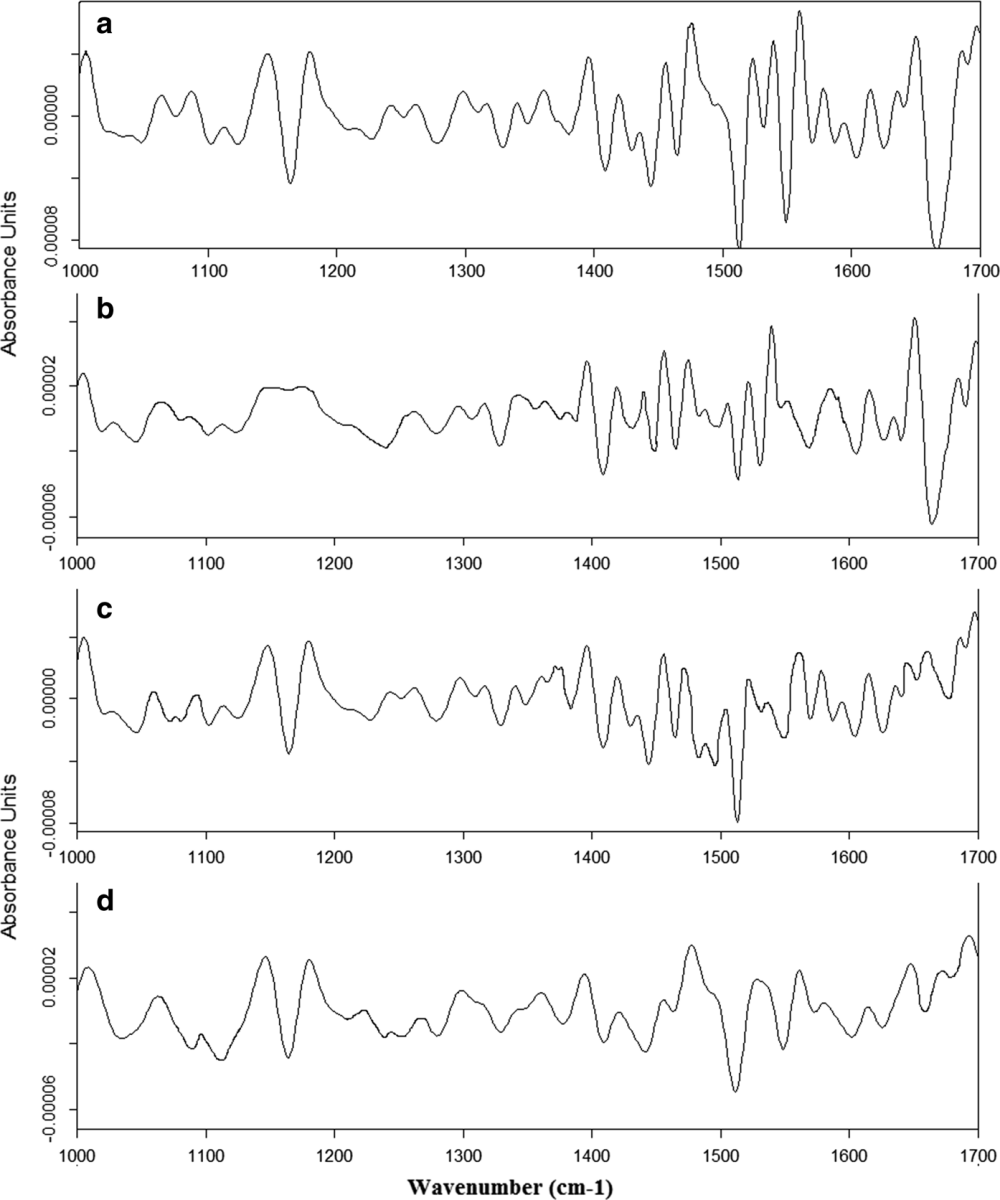
Second-derivative FTIR spectra of hazelnut pollen from: **a** Bieszczady (S1), **b** Krasne (S2), **c** Rzeszów (S3), **d** Zawiercie (S4)

**Fig. 5:**
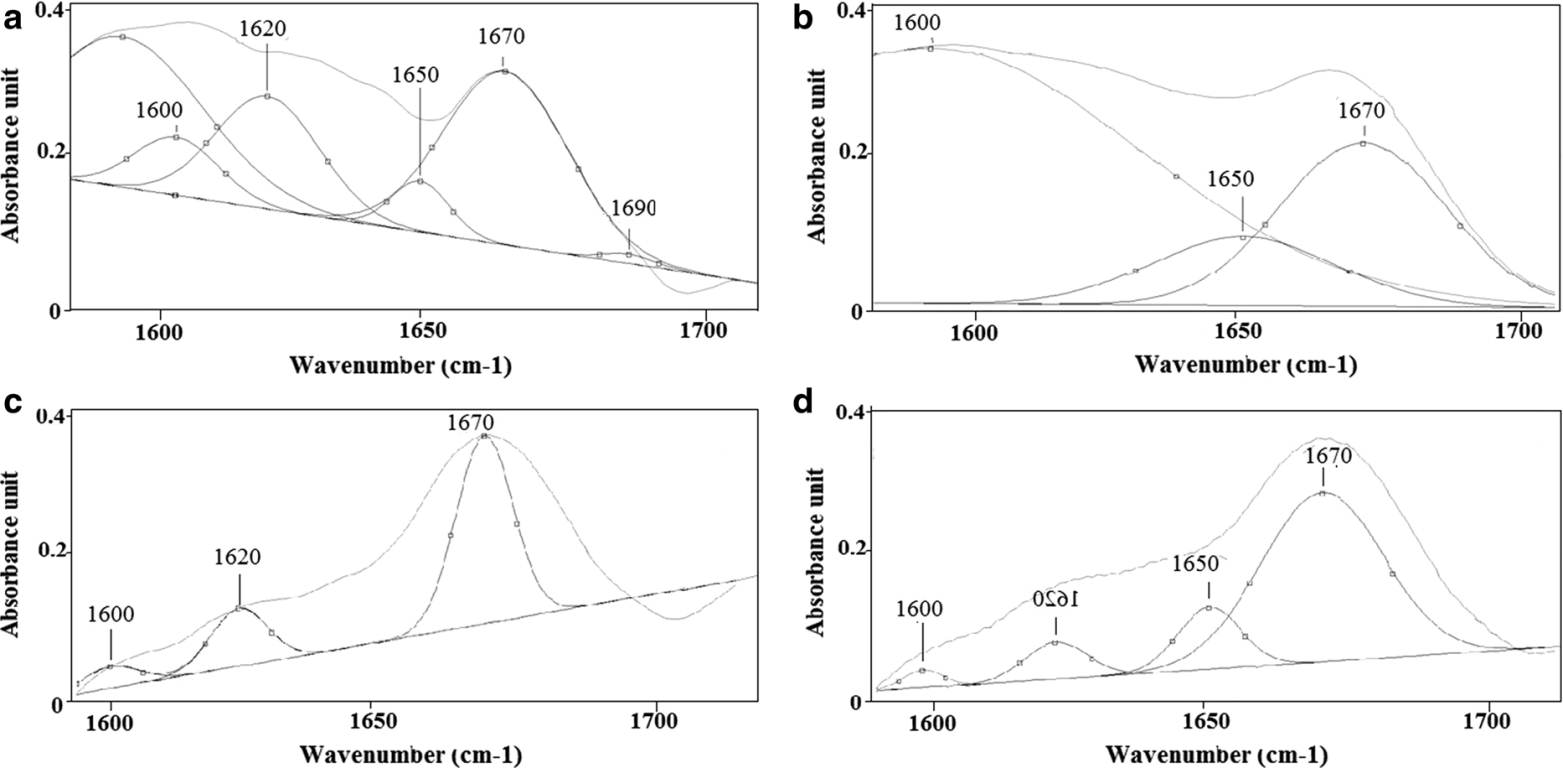
Curve-fitting analysis of the amide I profile of hazelnut pollen from: **a** Bieszczady (S1), **b** Krasne (S2), **c** Rzeszów (S3), **d** Zawiercie (S4)

In the second derivative of the spectra, structural changes in the regions corresponding to phospholipids, nucleic acid and proteins are visible. These changes were observed in the second derivatives of spectra from pollen samples collected in S2 (Fig. 4b), S3 (Fig. 4c) and S4 (Fig. 4d) compared to the one of the reference sample harvested in S1 (Fig. 4a). The more the other second-derivative FTIR spectra differ from that obtained for S1, the greater the change in the structure of molecules in this pollen. The most distinct changes are observed in the region corresponding to proteins in pollen from S4. Thus curve-fitting analysis of amide I profile was performed (Fig. 5) to obtain information about the type of secondary structural changes of α-helix and β-harmonica of proteins (Table 3).

**Table 3:** Wavenumbers with the corresponding vibrations of secondary structural changes in proteins (Dogan et al. 2007; Misra et al. 2015; Pandey et al. 2010; Mauerer and Lee 2006; Maury et al. 2005)

Wavenumber (cm^−1^)	Vibrations
1600	β-sheet
1620	β-sheet
1650	α-helisa
1670	β-turn
1690	β-turn

As the structural changes of proteins are visible only in the amide I region (1600–1700 cm^−1^), the curve-fitting analysis was performed only for this region, Fig. 5. Compared to the reference sample from S1 (Fig. 5a), in the spectrum from S2 (Fig. 5b), the absence of peaks corresponding to the secondary structure of proteins vibrations of β-sheet and β-turn (1620, 1690 cm^−1^, respectively) was observed. Furthermore, the pollen collected in S3 (Fig. 5c) did not exhibit oscillations assigned to α-helices (1650 cm^−1^) and β-turn (1690 cm^−1^). Moreover in the spectrum of pollen collected in S4 (Fig. 5d), the absence of the peak 1690 cm^−1^ (β-turn) is observed.

The number of the individual functions determines the secondary structure of proteins. Urbanization and pollution affect the formation of a particular secondary structure, and consequently, the function of proteins, Table 4. Between the samples, the percentages of the various structures are different. The pollen collected in S2 shows the highest percentage values of β-sheet (peak at 1600 cm^−1^) compared to the other pollen samples, especially to S4. A large percentage difference between the amounts of protein’s secondary structures is also observed in the case of the peak at 1670 cm^−1^. The largest variation is visible between pollen collected in S3 and samples collected in S1, S2 and S4.

**Table 4:** Curve-fitting analysis of protein’s secondary structures of hazel pollen collected from Bieszczady (S1), Krasne (S2), Rzeszów (S3), Zawiercie (S4)

Values [%]				
Wavenumber (cm^−1^)	Sites			
	Bieszczady S1	Krasne S2	Rzeszow S3	Zawiercie S4
1600	32	54	20	10
1620	21	Not present	20	21
1650	11	24	Not present	26
1670	25	22	60	43
1690	11	Not present	Not present	Not present

## Discussion

Plants react to soil and air pollution, urbanization, weather and environmental conditions, which can influence the pollen allergenicity (Lázaro et al. 2014; Todea et al. 2013; Koenig and Tabb 1980). The pollen collection sites exhibited different concentration and type of air pollutants and degree of urbanization. The most contaminated region was S4, followed by S3, S2 and the S1. In S4 the concentrations of air pollutants, especially PM10 and ozone, are higher than in S3, S2 and S1 Table 1 (www.katowice.pios.gov.pl/monitoring/informacje/stan2014/13ocena.pdf; www.wios.rzeszow.pl/cms/upload/edit/file/opracowania/jakosc_powietrza/2014/tekst_ocena_2014.pdf). Due to the presence of the steel mine and iron foundries the degree of urbanization in S4 also surpasses the development of urbanization in S3 or S2.

SEM imaging did not show any structural changes in pollen induced by anthropogenic factors. Quantitative and qualitative changes in hazel pollen under the influence of air pollution and urbanization were thus investigated by FTIR spectroscopy with curve-fitting analysis of amide I profile. Although no morphological changes in the pollen SEM images are visible, FTIR spectroscopy reveals important changes in the chemical composition of the pollen samples collected from different regions. These changes can be caused by air pollution (Dell’Anna et al. 2009; Cortegano et al. 2004; Armentia et al. 2002) or the urbanization rate (Asher 2011). It can be seen on the FTIR spectrum of the pollen collected in S2 (Fig. 3b) that the peak at 1170 cm^−1^, which corresponds to the asymmetrical vibration of the CO–OC group derived from glycogen, is absent. This can result from high concentrations of ozone (O_3_) or others factors. Although the accumulated amount of ozone (AOT40) does not exceed the threshold value for plants, it was higher in S3 than S1. According to the literature data, ozone as one of the products of fuel combustion can cause oxidation of amino acids, proteins and nucleic acids (Pasqualini et al. 2011). Moreover, the modification of the cell membrane under the influence of ozone is the main factor leading to secondary DNA damage and ultimately to cell death (Margalit et al. 2001). Ozone can therefore be at the origin of the qualitative changes of the pollen collected in S2, as shown in Fig. 3b.

In the FTIR spectra (Fig. 3) differences in absorbance between the pollen collected from S1 (Fig. 3a) and S2 (Fig. 3b) and the pollen collected from S3 (Fig. 3c) and S4 (Fig. 3d) can be observed. The quantitative changes of individual functional groups building the pollen molecule can result from different environmental factors typical for the place where the material was collected. From Table 1 it can be deduced that the concentrations of various pollutants (except ozone) are much higher in S3 and S4 compared to S2 and S1. Zimmermann et al. pointed that environmental factors such as dry heat stress or extremely rainy weather occurring during microsporogenesis, can influence the physiological processes and molecular composition of pollen of the same species. However, these changes concerned mainly nutrient composition and lipids, but not carbohydrates and proteins (Zimmermann and Kohler 2014).

The second derivative of the FTIR spectra (Fig. 4) shows that the major structural changes in pollen samples were found in the material collected from S4 (Fig. 4d) compared to the one from S1 (Fig. 4a). These changes can be caused by high concentrations of air pollutants, which in most cases exceed the norm for O_3_, benzo(A)pirene, PM10 and PM2.5. These contaminants directly affect the cells, which build the pollen and can cause mutations in the genetic material leading to changes in structure and chemical properties of the pollen molecule (Dell’Anna et al. 2009). Structural changes are more visible in the curve-fitting analysis of the amide I profile. Indeed, in each of the pollen samples collected from S2, S3 or S4—Fig. 5b, c or d, respectively, compared with the material collected from S1 (Fig. 5a), some peaks corresponding to the secondary structure of proteins were absent. Furthermore, quantification of the α-helices and β-harmonica also differ between respective tested pollen. It could be caused among others by environmental factors.

## Conclusions

The results indicate that anthropogenic stress has an impact not only on quantitative changes (different absorbance values of individual functional groups, which build the chemical structures of pollen), but also qualitative changes (in the secondary structure of proteins). The structural changes in proteins, observed in the second derivative of the FTIR spectrum and the curve-fitting analysis of amide I profile, result from mutations occurring in the genetic material, which can be caused inter alia by air pollutants. This can lead to more aggressive allergenic proteins and hence to more frequent cases of allergy in people.

Concluding, FTIR spectroscopy is a sensitive indicator of chemical compounds such as nucleic acid, proteins, polysaccharides and lipids constituting the *C. avellana* pollen. Moreover, the FTIR technique in combination with curve-fitting analysis allows determining the secondary structure of proteins and thus can also identify the changes in proteins induced by different factors such as pollutants. Consequently, this method can be recommended to investigate biological materials, as it allows to identify structural and chemical changes in the pollen grains.
